# Exploring 5-MeO-DMT as a pharmacological model for deconstructed consciousness

**DOI:** 10.1093/nc/niaf007

**Published:** 2025-04-21

**Authors:** Christopher Timmermann, James W Sanders, David Reydellet, Tommaso Barba, Lisa X Luan, Óscar Soto Angona, Genís Ona, Giancarlo Allocca, Carl H Smith, Zachary G Daily, Natasha L Mason, Lilian Kloft-Heller, Martin Kuchar, Lucie Janeckova, Tomas Palenicek, David Erritzoe, Johannes G Ramaekers, Robin L Carhart-Harris, Malin Vedøy Uthaug

**Affiliations:** DMT Research Group, Centre for Psychedelic Research, Department of Brain Sciences, Imperial College London, London W12 0NN, UK; Department of Experimental Psychology, University College London, London WC1H 0AP, UK; DMT Research Group, Centre for Psychedelic Research, Department of Brain Sciences, Imperial College London, London W12 0NN, UK; DMT Research Group, Centre for Psychedelic Research, Department of Brain Sciences, Imperial College London, London W12 0NN, UK; DMT Research Group, Centre for Psychedelic Research, Department of Brain Sciences, Imperial College London, London W12 0NN, UK; DMT Research Group, Centre for Psychedelic Research, Department of Brain Sciences, Imperial College London, London W12 0NN, UK; Parc Sanitari Sant Joan de Dèu, Barcelona 08830, Spain; Department of Psychiatry and Forensic Medicine, Universitat Autònoma de Barcelona, Barcelona 08193, Spain; Parc Sanitari Sant Joan de Dèu, Barcelona 08830, Spain; Fundació Sant Joan de Déu, Barcelona 08014, Spain; Medical Anthropology Research Center (MARC), Universitat Rovira i Virgili, Tarragona 43005, Spain; Florey Department of Neuroscience and Mental Health, The University of Melbourne, Parkville, Victoria 3052, Australia; Somnivore Pty Ltd, Melbourne, Victoria 3340, Australia; Department of Media, School of the Arts and Creative Industries, University of East London, London, E16 2RD, UK; DMT Research Group, Centre for Psychedelic Research, Department of Brain Sciences, Imperial College London, London W12 0NN, UK; Department of Neuropsychology and Psychopharmacology, Faculty of Psychology and Neuroscience, Maastricht University, 6229, the Netherlands; Department of Neuropsychology and Psychopharmacology, Faculty of Psychology and Neuroscience, Maastricht University, 6229, the Netherlands; Forensic Laboratory of Biologically Active Substances, Department of Chemistry of Natural Compounds, University of Chemistry and Technology, Prague 166 28, Czech Republic; Forensic Laboratory of Biologically Active Substances, Department of Chemistry of Natural Compounds, University of Chemistry and Technology, Prague 166 28, Czech Republic; Psychedelic Research Center, National Institute of Mental Health, Klecany 250 67, Czech Republic; 3rd Faculty of Medicine, Charles University in Prague, Prague 100 00, Czech Republic; DMT Research Group, Centre for Psychedelic Research, Department of Brain Sciences, Imperial College London, London W12 0NN, UK; Department of Neuropsychology and Psychopharmacology, Faculty of Psychology and Neuroscience, Maastricht University, 6229, the Netherlands; DMT Research Group, Centre for Psychedelic Research, Department of Brain Sciences, Imperial College London, London W12 0NN, UK; Department of Neurology, University of California San Francisco, San Francisco 94143, USA; DMT Research Group, Centre for Psychedelic Research, Department of Brain Sciences, Imperial College London, London W12 0NN, UK; Somnivore Pty Ltd, Melbourne, Victoria 3340, Australia; Department of Neuropsychology and Psychopharmacology, Faculty of Psychology and Neuroscience, Maastricht University, 6229, the Netherlands

**Keywords:** psychedelic, serotonin, EEG, self, awareness, neurophenomenology

## Abstract

5-MeO-DMT is a short-acting psychedelic that is anecdotally reported to induce a radical disruption of the self and a paradoxical quality of aroused, waking awareness that is nevertheless devoid of any specific perceptual contents. Here, we conducted an exploratory observational study of the phenomenological and neuronal effects of this compound. We collected micro-phenomenological interviews, psychometric questionnaires, and electroencephalography (EEG) in naturalistic ceremonial settings where 5-MeO-DMT was ingested. Results revealed that the 5-MeO-DMT experience followed a dynamic progression that—only in the most extreme cases—manifested as a complete absence of self-experience and other phenomenal content with preserved awareness. Furthermore, visual imagery, bodily self-disruption, narrative self-disruption, and reduced phenomenal distinctions occurred in a variable fashion. EEG analyses revealed the 5-MeO-DMT experience was characterised by (global) alpha and (posterior) beta power reductions, implying a mode of brain functioning where top-down models are inhibited. Our preliminary phenomenological findings confirm the potential utility of 5-MeO-DMT as a pharmacological model for deconstructed consciousness while noting the limitations of employing retrospective questionnaires for this purpose. Considering the exploratory nature of this study and its limitations inherent to its naturalistic nature, further research employing real-time experience sampling and phenomenologically trained participants in controlled environments could expand our findings to meaningfully inform the potential of this tool for the scientific study of consciousness.

## Introduction

Psychedelics are compelling tools for the study of consciousness, as they induce significant alterations in the contents of conscious experience while preserving wakefulness at commonly used doses (Timmermann et al. 2023a). 5-MeO-DMT is a classic, psychedelic compound which acts via agonism at the serotonin-2A receptor (Nichols 2016). However, it is atypical in comparison to other classic psychedelics, as preliminary reports suggest an especially potent and rapid disruption in self-related processing (Uthaug et al. 2019) while inducing comparatively less visual imagery, which may relate to its unusually high agonism at the serotonin-1A receptor (Ermakova et al. 2022).

The subjective effects of 5-MeO-DMT make it a promising molecule for the study of human consciousness, as it may not only reliably induce experiences of self-dissolution but also, in peak moments of intensity, lead to a further deconstruction of all contents of experience, including thoughts and perception, with wakefulness preserved. This is anecdotally described as a ‘void’ experience (Metzner 2013). These experiences resonate with the phenomenology of so-called ‘nonduality’ that has been reported in some forms of meditation. Such states have been proposed as an avenue to study the minimal constituents of subjectivity or a ‘minimal phenomenal experience’ (MPE; Metzinger 2020). Compared to meditation states, however, the administration of 5-MeO-DMT might represent an attractive alternative to study states of deconstruction in a controlled fashion. This is because studying such extreme conscious states using meditation might require participants with rare, advanced skills that take considerable time and training to acquire. Furthermore, when 5-MeO-DMT is inhaled, the onset of effects occur within seconds, and the resolution of these effects tends to resolve within 30 minutes (Metzner 2013), making this compound a useful tool to study the deconstruction of conscious experience.

While the pharmacological induction of deconstructed consciousness represents a significant opportunity for the neuroscience of consciousness, it is imperative first to determine to what extent 5-MeO-DMT experiences truly reflect these target states. Intense psychedelic experiences are particularly susceptible to reporting artefacts due to their ineffable character and the potential role of confabulation in subsequent descriptions (Timmermann et al. 2022). For researchers to meaningfully employ psychedelics in the scientific study of consciousness, innovative methods must be implemented to address these limitations. Rigorous first-person descriptions of subjective experiences, such as those produced during micro-phenomenological interviews (MPIs), have been found to quantitatively improve commonly reported issues associated with introspection and recollection (Petitmengin et al. 2013), and have become increasingly attractive in neurophenomenological studies linking subjective experiences of non-ordinary states of consciousness and brain activity (Timmermann et al. 2023a).

In this study, we performed a preliminary neurophenomenological naturalistic investigation of 5-MeO-DMT. We measured the brain activity of healthy volunteers using electroencephalography (EEG) before and during the administration of 5-MeO-DMT in naturalistic settings, followed by MPIs and psychometric questionnaires. We hypothesised that 5-MeO-DMT disrupts several features of the sense of self as well as the power of alpha brain rhythms.

## Materials and methods

### Participants

Participants in this observational study were recruited based on their intention to participate in a ceremony where psychedelics would be ingested. For this study, we focused on individuals planning to participate in a ceremony where either synthetic 5-MeO-DMT or parotoid gland secretions from the *Incilius alvarius* toad would be ingested (the main psychoactive component of these secretions is 5-MeO-DMT; Uthaug et al. 2019). Additional eligibility criteria included being 18 years or older and having a good understanding of English. Ethics approval was given by the Joint Research Compliance Office, the Imperial College Research Ethics Committee (ICREC reference 18IC4346), and the Ethical Review Committee Psychology and Neuroscience (ERCPN), Maastricht University. All participants gave informed consent.

### Procedure

For this study, we collected data from participants in ceremonies occurring in the Netherlands and Spain. The research team was not involved with the organisation of the ceremonies or administration of 5-MeO-DMT. In both ceremonies, 5-MeO-DMT (either synthetic or from toad secretion) was inhaled, via a pipe, by participants assisted by a facilitator. The sample consisted of 14 participants (5 females, 8 males, average age 35.8 ± 11.8; 4 participants failed to complete data relative to age, and 1, to gender). Synthetic 5-MeO-DMT was used in the Netherlands ceremony (43% of participants), and toad secretions were used in the Spain ceremony (57% of participants). The facilitator of each ceremony chose the doses. The average dose for the Netherlands ceremony was 15.4 mg of 5-MeO-DMT. Chemical analyses from the Spain ceremony revealed the average dose to be 28.7 mg. In the latter ceremony, participants inhaled from multiple preprepared pipes (EEG data were analysed only after the last inhalation), allowing for the comparatively higher dose of 5-MeO-DMT (Supplementary methods and Supplementary Table S1 for details).

### Phenomenology

Following our previous work (Timmermann et al. 2019), MPIs were conducted (by author C.T. for the Netherlands and Spain ceremonies and J.W.S. for the Spain ceremony), immediately upon return to baseline following the administration of 5-MeO-DMT. The MPIs were audio-recorded, then transcribed, and analysed (by J.W.S.) to capture time-dependent phenomenological features of the experience. The interviews lasted approximately 30–60 min. The MPI is beneficial to explore qualities of the experience which may go commonly unnoticed (Petitmengin 2006), and is shown to be helpful in reducing confabulation (Petitmengin et al. 2013) and the influence of posthoc interpretations of ineffable aspects of experience commonly encountered in non-ordinary states of consciousness (Timmermann et al. 2023a). In each interview, the trained interviewer guided the participant to immerse themselves in the memory of the 5-MeO-DMT experience and give a rich and systematic description of how it unfolded from moment to moment. Through an iterative line of questioning, the participant was invited to describe their experience in ever-more precise detail, focusing on the acute phenomenological qualities and structures while avoiding extraneous details such as posthoc explanations and judgements of the experience. Transcripts of the interviews were subjected to a combined micro-phenomenological and thematic analysis (Clarke and Braun 2017, Petitmengin et al. 2018), whereby micro-phenomenological analysis was used to parse each interview into a timeline of phenomenological phases according to linguistic indicators of temporal shifts, and thematic analysis was used to cluster these phases across participants according to the phenomenological characteristics described within each. These clusters formed categories that represent a distinct substate of the 5-MeO-DMT experience. Assessing transitions between these categories in the phase timeline of each interview supported the development of a flowchart model describing the pathways followed through the identified substates. The Altered States of Consciousness Questionnaire (ASC), a self-report retrospective measure lacking any temporal assessments, was completed after the interview to complement phenomenological findings (Studerus et al. 2010).

### Electroencephalography

Brain activity was recorded continuously from baseline (for 5 min) as well as during and after 5-MeO-DMT administration (for up to 15 min) using a 24-channel wireless EEG cap (DSI-24 System, Wearable Sensing) with 21 dry electrodes (300 Hz sampling rate). Participants remained in a seated position for all recordings with their eyes closed. Electrodes were positioned following the 10–20 international format with electrode Pz for online reference, and earlobe electrodes A1 and A2 to subtract noise in other channels. Data were recorded using a Bluetooth-connected DSI-Streamer-v.1.08.41 and processed using the Fieldtrip toolbox (Oostenveld et al. 2011) in MATLAB. Data were demeaned, bandpass filtered (1–30 Hz), and visually inspected for removal of gross artefacts. Independent Component Analysis was then employed, and components displaying electrocardiogram activity or ocular motion were removed. Five participants were removed from further analysis as no usable data survived preprocessing. Spectral analysis was performed using Hanning windowed Fast Fourier Transforms between 1 and 30 Hz at 0.5 Hz intervals, and averaged in delta (1–4 Hz), theta (4–8 Hz), alpha (8–13 Hz), low beta (13–20 Hz), and high beta (20–30 Hz) bands. Gamma power and entropy metrics known to relate to fast rhythms (Mediano et al. 2023) are highly susceptible to muscle artefacts and were thus not included in analyses. Statistical analyses were performed by comparing power at each frequency band obtained in the 5-min immediately after administration against the 5-min baseline by performing cluster-based *t*-statistics testing (Oostenveld et al. 2011). Considering the sample size and variability of the data, a qualitative neurophenomenological analysis was employed to examine changes in brain activity (z-scored) of participants reporting a complete absence of the self compared to participants reporting some preservation of features of the self.

## Results

### Micro-phenomenology

**Figure 1:**
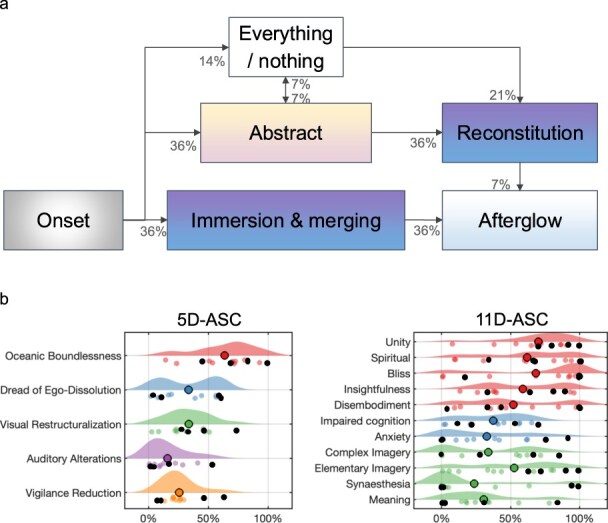
Characterisation of the 5-MeO-DMT experience. (a) Analysis of interviews following 5-MeO-DMT resulted in six categories of experience. Arrows and percentages describe the temporal progression between categories and their frequency of occurrence (initial transitions do not add to 100% because progressions were not identified for all participants). (b) Results from self-reported retrospective ASC questionnaire showing scores for the 5 (left) and 11 (right) dimensions (colours in the 11 dimensions are matched to their grouping in the 5 dimensions). These subscales revealed high variability in a wide range of phenomenological features, including for participants experiencing the ‘everything/nothing’ stage (shown as black dots), which are distributed in a wide range of scores (with the exception of the ‘Unity’ dimension), reflecting the inherent limitations of static, self-reported measures (Large coloured dots represent the mean).

Six categories characterising distinct phases of the 5-MeO-DMT experience were identified. Each was visited by a subset of the participants at some point. A description of each category is given below, along with the percentage of participants who reported it. The direction and frequency of transitions between categories were also identified (Fig. 1a).

### Onset (86%)

The onset of the experience affected different modalities and dimensions of experience. The dynamic of ‘onset’ was rapid and variously described as ‘fading’, ‘accelerating’, ‘expanding’, ‘compressing’, ‘tearing’, ‘fractalising’, ‘dissolving’, ‘shattering’, or ‘collapsing’. These were described as affecting the body, the visual field, the thinking mind, the self, or all of the above—with each potentially being affected by different processes (e.g. expanding body and shattering visuals).

“At the very beginning, it was like something was crumbling … like, disintegrating … It was black. But I saw these … patterns … as if there was a pattern that was somehow crumbling, and it opened up to something big and enormous. And that was very quick.” P14

### Immersion/merging (43%)

Described as a reduction in awareness of immediate surroundings, accompanied by intense feelings moving through, or being released from, the body. Sometimes with a described sense of ‘becoming’ the feelings rather than just feeling them, or with sensations that are in between or beyond the senses. In some cases, feelings were identified as emotions, and at times those emotions were experienced as depersonalised (i.e. not connected to one’s own personal narrative context). Feelings were described as either positive (blissful, sensual) or negative (fear, grief, discomfort). Sometimes, visuospatial language such as colour and shape was used to describe the state; however, in some cases this was qualified as being not truly visual.

“I could feel energy centred in the torso … warm energy … very beautiful … The emotions are giving birth to energy within you … I was feeling like I’m letting go of something … I didn’t really exist as a person … my surroundings didn’t exist … like I was the emotions and the energy.” P2

### Abstract (43%)

A disembodied experience in which some elementary visuospatial form was described, using terms such as shape, colour, and movement. However, despite these visuospatial terms, the experience was sometimes further described as lacking spatial, visual, and also temporal dimensions. The form was sometimes described as being experienced through something akin to bodily sensations, but not in the everyday form of body at all (i.e.: felt sensations, but not feelings of the body). No other contents were described, including no narrative self and no capacity for thought or reflection.

“There were no limits, or no objects, no dimensions even. I had no thought. I had no senses … But I had some feeling of … like a pyramid or something … And the colour … it was kind of a rose [colour]. [But] it is not that I saw this … it was more kind of feeling of the space … So there’s a feeling of this kind of rose-coloured pyramid.” P5

### Everything/nothing (29%)

A state of extreme reduction in phenomenological distinctions such that, relatively, very little was described, and what was described was often done so in paradoxical terms. It featured no spatial or temporal qualities, no distinct sensory content, no capacity for thought or reflection, no narrative or bodily self, and no subject-object dichotomy. The experience was sometimes described as ‘whole’ or ‘full’, containing everything, despite a concurrent sense of ‘void’ or ‘nothing’. It was sometimes described as luminous, or with a positive affective tone, though still lacking the structures of perception and selfhood that are implied by categories like vision and emotion.

“I was not there. It was like I was accessing ‘whiteness’. Quiet, blissful, enormous, whiteness … I was everything … There was no controller, or observer … It was not ‘me’. I don’t know what happened. I somehow had a glimpse of, of … of the wholeness.” P6

### Reconstitution (57%)

A gradual and sequential return of structures of experience, and sensory experience of the present environment. The order in which specific structures and content returned was variable across participants. An example sequence could be as such: sounds return first, followed by bodily sensations, followed by a broader awareness of the room, followed by thoughts and narrative self. Upon return, structures or content were sometimes described as distant or faint before gradually becoming clearer and more present. A rapid or discontinuous reconstitution was also described in some cases.

“I started feeling my shoulders … it was a bit subtle, and I wasn’t sure if [the feeling] was actually true … And then I was puzzled … like, ‘why are there people [here]?’ … This is where the duality started to happen. Like, that is me. And, that is the space. And, there are other things.” P9

### Afterglow (43%)

A state close to baseline consciousness but with a lingering sense of clarity, positive affect, and/or reduction in thought. Sometimes described as a meditative or peaceful state.

“[It was] like a meditation. Just ‘feel’, and just ‘be’. … the quality of my presence feels different … like I’ve been stretched out a bit.” P13

### Memory effects

Some participants described moments of the experience as being impossible or very difficult to recall, possibly related to amnesia or losing consciousness. This phenomenon was temporally associated with peak phases of the experience and exclusively by participants from the Spain ceremony (63%), who received higher doses. Therefore, categories such as ‘abstract’ and ‘everything/nothing’ may be underreported due to amnesia (see Supplementary Table S3 for the occurrence of phenomenological categories for each ceremony).

## Questionnaire results

Average scores of the ASC questionnaire were variable across participants, including for those reporting peak experiences (i.e. the ‘everything/nothing’ category), possibly reflecting the limitations of self-report questionnaires lacking temporal precision (Fig. 1b). Further highlighting this point, whereas average scores of the Oceanic Boundlessness subscale—closely related to peak experiences of interest (Roseman et al. 2018)—were, on average, higher for the Netherlands ceremony (71.3% of total) compared to the Spain ceremony (58.7%) (Supplementary Fig. S1), the MPI analyses identified peak experiences occurring more frequently in the Spain ceremony (Supplementary Table S3), where a larger average dose of 5-MeO-DMT was used (however, it is important to note that nonpharmacological variables related to the setting of the ceremony may have also influenced this difference) (Supplementary Table S2).

The phenomenological categories were then associated with five broadly studied structures of experience in consciousness science, with results suggesting that ‘abstract’ and ‘everything/nothing’ experiences hint to a state of deconstructed consciousness (Table 1).

**Table 1.** Phenomenological categories of the 5-MeO-DMT experience as they typically relate to five broadly studied structures of experience

	Embodied self	Narrative self	Thought/reflection	Disconnection	Phenomenal distinctions
Onset	Present, undergoing transformation	Present, undergoing transformation	Present, undergoing transformation	Connected or in process of disconnection	Many
Immersion and Merging	Present with intense sensations, or reduced	Reduced	Reduced	Partially disconnected	Reduced
Abstract	Absent, or transformed beyond recognition	Absent	Absent	Disconnected	Few
Everything/Nothing	Absent, or universal and featureless	Absent	Absent	Disconnected	Minimal
Reconstitution	Transitioning from absent to present	Transitioning from absent to present	Transitioning from absent to present	Transitioning from disconnected to connected	Transitioning from minimal or few towards baseline
After(glow)	Present or slightly reduced	Present or slightly reduced	Present or slightly reduced	Connected	As per baseline or slight reduction

### Electroencephalography

**Figure 2:**
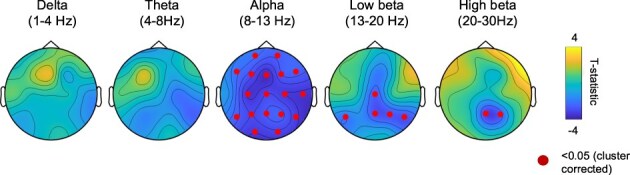
EEG effects of 5-MeO-DMT. Ingestion of 5-MeO-DMT resulted in global reductions of alpha power across most electrodes, and decreases in posterior beta band power.

EEG results revealed that, compared to baseline, the 5-MeO-DMT state was associated with a global reduction of power in alpha brainwaves (max. t[9] = 3.77, *P* = .0001, cluster-corrected for multiple comparisons) and a posterior decrease in low (max. t[9] = *P* = .007, cluster-corrected for multiple comparisons) and high beta power was also observed (max. t[9] = 3.18, *P* = .021, cluster-corrected for multiple comparisons) (Fig. 2).

### Neurophenomenology

**Figure 3:**
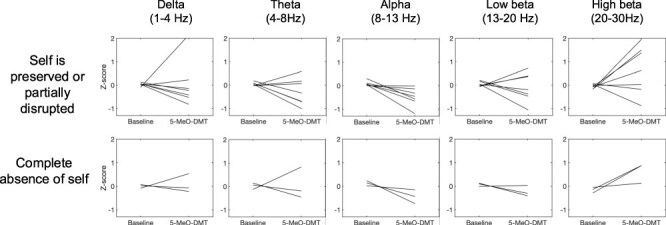
Relationship between self-disruption and EEG spectra. Qualitative inspection of EEG spectra of participants reporting a complete absence of the self (for which the ‘abstract’ or everything/nothing’ stages were identified) did not show any overt differences compared to participants who reported some preservation of the self.

To explore the impact of 5-MeO-DMT on the self, we inspected changes in EEG spectra separately for participants for which the ‘abstract’ and ‘everything/nothing’ categories were identified, as in these categories embodied and narrative features of the self were completely absent, compared to other participants for which only categories, where the self was preserved or only partially absent, were identified. Qualitative inspection of the spectra did not show a discrepancy between these groups. However, it is important to note that it was not possible to estimate when specific phenomenology corresponded to specific changes in brain activity within each subject as no real-time experience sampling was performed to estimate such changes and only averaged EEG activity throughout the whole period was used for neurophenomenological analyses (Fig. 3).

## Discussion

In this exploratory, observational study, we investigated the effects of 5-MeO-DMT on experience and brain activity, based on the premise that 5-MeO-DMT may represent a useful model for deconstructed consciousness. Consistent with this notion, our results showed that peak 5-MeO-DMT experiences resulted in a state of disconnection, loss of narrative and embodied aspects of the self, and minimal phenomenal distinctions, while preserving wakefulness. However, these experiences occurred in approximately a third of participants and at specific moments only. EEG findings showed that a broad inhibition of alpha and posterior beta power was associated with the administration of 5-MeO-DMT.

Our phenomenological findings suggest that, during peak moments, 5-MeO-DMT induced a state of deconstructed consciousness. This state was not just linked to reductions in the embodied and narrative selves but also presented a state of heightened disconnection from the environment and (at times) associated with heightened levels of amnesia. Nonetheless, we found this state to be present only for some participants and at specific moments of the experience, something that could not be identified with the commonly used ASC questionnaire. A higher prevalence of this state among participants with larger doses indicates that the state may be dose-dependent, though amnesic effects may be similarly dose-dependent, and a dosing ‘sweet spot’ between the two may exist. Furthermore, these analyses revealed the 5-MeO-DMT state to be dynamic, with phenomenological trajectories varying between individuals. It is possible that this variability hindered the ability of neurophenomenological analysis to pick up on differences in brain activity of participants reporting the full absence of self, as opposed to participants reporting some preservation of features of the self. These results are consistent with the notion that temporal- and phenomenologically-guided analyses are required to establish the brain states associated with these experiences of deconstruction, which hold significant relevance to the study of self-experience and consciousness more broadly (Timmermann et al. 2023a). Future studies employing real-time sampling of experiences of interest are particularly relevant to detect the neural correlates of these states, especially considering the amnesic components of the 5-MeO-DMT experience that we report here.

In further support of temporal- and phenomenologically-guided analyses, self-report questionnaire results reflected somewhat counterintuitive differences in dosages of 5-MeO-DMT (synthetic vs. toad secretion) between the two ceremonies. Micro-phenomenological interviews more adequately captured these. We have established this sensitivity of micro-phenomenology to neurobiologically relevant dimensions of psychedelic experience in previous work (Timmermann et al. 2019); however, further work in a controlled lab setting should seek to validate these 5-MeO-DMT-specific phenomenological categories and potential dose-dependent effects against neurobiological data.

EEG findings of reduced alpha power are consistent with those of other psychedelics and dissociatives (Ona et al. 2022, Erritzoe et al. 2024). Alpha power has been linked to the encoding of high-level models (such as those related to the self), and thus, psychedelic-induced reductions have been linked with the reduced influence of these models on brain function (Carhart-Harris and Friston 2019). Our results suggest a similar inhibition of these models may also occur during the 5-MeO-DMT experience. Results of reduced posterior beta power under 5-MeO-DMT also resonate with similar decreases following the administration of DMT (Timmermann et al. 2019, 2023b), which correlate with bodily self-disruption (Timmermann et al. 2019). Intriguingly, disruptions of alpha and posterior beta power have also been found in the dissolution of bodily boundaries experienced during advanced meditation, with beta power reductions localised to the posterior cingulate cortex (Trautwein et al. 2024), a hub of the Default Network, which has a central role in self-related processing (Yeshurun et al. 2021). Apart from differences in these two bands, a high variability of effects was seen for other frequency bands. This variability resonates with contradictory results from different psychedelics on delta, theta, and gamma bands (Timmermann 2020). It is possible that such variability reflects phenomenological differences across subjects or variability in drugs, dose, or experimental setting. Future studies are required to establish the role of these variables in brain function.

Due to the uncontrolled environment of our study, we chose not to examine the effects of 5-MeO-DMT in brain entropy (see ‘Methods’ section). Entropy has been hypothesised to index the richness of an experience (Carhart-Harris et al. 2014). It is plausible that the increase in richness of contents in a high-intensity psychedelic experience (such as the one induced by 5-MeO-DMT) is such that it surpasses the attentional capacity required to perceive these in an organised fashion, and that this results in such minimal phenomenal distinctions we report here. Our phenomenological results seem to support such a hypothesis where participants recall a paradoxical experience of ‘everything and nothing’. If this is correct, then such deconstructed experiences induced by 5-MeO-DMT would be indexed by an increase in neuronal entropy rather than a decrease.

Testing this neurophenomenological hypothesis requires employing both phenomenologically-guided analyses as well as adequate controls for EEG confounds. Increases in power of low and high brainwaves have been reported following naturalistic and lab reports of 5-MeO-DMT and - the closely-related compount - DMT (Acosta-Urquidi 2017, Pallavicini et al. 2021, Timmermann et al. 2023b, Blackburne et al. 2024). To confirm such reports (as well as our current EEG findings), it is imperative to control for potential physiological artefacts, especially considering that these compounds markedly increase cardiovascular and muscle-related artefacts known to impact measurement of these brain mechanisms.

It is relevant to note some limitations in our study. The naturalistic design of our study and relatively low sample prevented us from having experimental controls or performing experience sampling, which would have allowed us to refine the association between phenomenology and brain activity. Moreover, we averaged data from participants ingesting synthetic and natural sources of 5-MeO-DMT for EEG analyses. Future studies should aim for larger samples and would benefit from employing real-time experience sampling in controlled environments.

## Supplementary Material

niaf007_Supp

## Data Availability

Data from this manuscript are available upon reasonable request to the corresponding author.

## References

[R1] Acosta-Urquidi J . EEG studies of the acute effects of the visionary tryptamine DMT. In: Columbus AM (ed.), *Advances in Psychology Research*. New York: Nova Science Publishers, 2017, 173–200.

[R2] Blackburne G, McAlpine RG, Fabus M et al. Complex slow waves radically reorganise human brain dynamics under 5-MeO-DMT. 2024. doi: 10.1101/2024.10.04.616717 40700019

[R3] Carhart-Harris RL, Friston KJ. REBUS and the anarchic brain: toward a unified model of the brain action of psychedelics. *Pharmacol Rev* 2019;71:316–44. doi: 10.1124/pr.118.017160 31221820 PMC6588209

[R4] Carhart-Harris RL, Leech R, Hellyer PJ et al. The entropic brain: a theory of conscious states informed by neuroimaging research with psychedelic drugs. *Front Hum Neurosci* 2014;8:1–22. doi: 10.3389/fnhum.2014.00020 24550805 PMC3909994

[R5] Clarke V, Braun V. Thematic analysis. *J Posit Psychol* 2017;12:297–8. doi: 10.1080/17439760.2016.1262613

[R6] Ermakova AO, Dunbar F, Rucker J et al.. A narrative synthesis of research with 5-MeO-DMT. *J Psychopharmacol* 2022;36:273–94. doi: 10.1177/02698811211050543 34666554 PMC8902691

[R7] Erritzoe D, Timmermann C, Godfrey K et al.. Exploring mechanisms of psychedelic action using neuroimaging. *Nat Mental Health* 2024;2:141–53. doi: 10.1038/s44220-023-00172-3

[R8] Mediano PA, Rosas FE, Luppi AI et al. Spectrally and temporally resolved estimation of neural signal diversity. *eLife* 2023;e88683.doi: 10.7554/eLife.88683.1

[R9] Metzinger T . Minimal phenomenal experience meditation, tonic alertness, and the phenomenology of. *Philosophy Mind Sci* 2020;1:1–44

[R10] Metzner R . The Toad and the Jaguar: A Field Report of Underground Research on a Visionary Medicine Bufo alvarius and 5-methoxy-dimethyltryptamine. Berkeley, CA: Regent Press, 2013.

[R11] Nichols DE . Psychedelics. *Pharmacol Rev* 2016;68:264–355. 26841800 10.1124/pr.115.011478 PMC4813425

[R12] Ona G, Sampedro F, Romero S et al.. The kappa opioid receptor and the sleep of reason: cortico-subcortical imbalance following salvinorin-A. *Int J Neuropsychopharmacol* 2022;25:54–63. doi: 10.1093/ijnp/pyab063 34537829 PMC8756086

[R13] Oostenveld R, Fries P, Maris E et al. Fieldtrip: open source software for advanced analysis of MEG, EEG, and invasive electrophysiological data. *Comput Intell Neurosci* 2011;2011:1–9. doi: 10.1155/2011/156869 21253357 PMC3021840

[R14] Pallavicini C, Cavanna F, Zamberlan F et al. Neural and subjective effects of inhaled N,N-dimethyltryptamine in natural settings. *J Psychopharmacol* 2021;35:406–20. doi: 10.1177/0269881120981384 33567945

[R15] Petitmengin C . Describing one’s subjective experience in the second person: an interview method for the science of consciousness. *Phenomenol Cogn Sci* 2006;5:229–69. doi: 10.1007/s11097-006-9022-2

[R16] Petitmengin C, Remillieux A, Cahour B et al. A gap in Nisbett and Wilson’s findings? A first-person access to our cognitive processes. *Conscious Cogn* 2013;22:654–69. doi: 10.1016/j.concog.2013.02.004 23719334

[R17] Petitmengin C, Remillieux A, Valenzuela-Moguillansky C. Discovering the structures of lived experience: towards a micro-phenomenological analysis method. *Phenomenol Cogn Sci* 2018;18:691–730. doi: 10.1007/s11097-018-9597-4

[R18] Roseman L, Nutt DJ, Carhart-Harris RL. Quality of acute psychedelic experience predicts therapeutic efficacy of psilocybin for treatment-resistant depression. *Front Pharmacol* 2018;8:974. doi: 10.3389/fphar.2017.00974 PMC5776504 29387009

[R19] Studerus E, Gamma A, Vollenweider FX. Psychometric evaluation of the altered states of consciousness rating scale (OAV). *PLoS One* 2010;5:e12412. doi: 10.1371/journal.pone.0012412 PMC2930851 20824211

[R20] Timmermann C . The Effects of DMT and Associated Psychedelics on the Human Mind and Brain. London: Imperial College London, 2020.

[R21] Timmermann C, Bauer PR, Gosseries O et al.. A neurophenomenological approach to non-ordinary states of consciousness: hypnosis, meditation, and psychedelics. *Trends Cognit Sci* 2023a;27:139–59. doi: 10.1016/j.tics.2022.11.006 36566091

[R22] Timmermann C, Roseman L, Haridas S et al.. Human brain effects of DMT assessed via EEG-fMRI. *Proc Natl Acad Sci* 2023b;120:e2218949120. doi: 10.1073/pnas.2218949120 PMC10068756 36940333

[R23] Timmermann C, Roseman L, Schartner M et al.. Neural correlates of the DMT experience assessed with multivariate EEG. *Sci Rep* 2019;9:16324. doi: 10.1038/s41598-019-51974-4 PMC6864083 31745107

[R24] Timmermann C, Watts R, and Dupuis D. Towards psychedelic apprenticeship: developing a gentle touch for the mediation and validation of psychedelic-induced insights and revelations. *Transcult Psychiatry* 2022;59: 691–704. doi: 10.1177/13634615221082796 35313754 PMC9660279

[R25] Trautwein FM, Schweitzer Y, Dor-Ziderman Y et al. Suspending the embodied self in meditation attenuates beta oscillations in the posterior medial cortex. *J Neurosci* 2024;44:e1182232024. doi: 10.1523/JNEUROSCI.1182-23.2024 PMC11211716 38760162

[R26] Uthaug MV, Lancelotta R, Szabo A et al. Prospective examination of synthetic 5-methoxy-N,N-dimethyltryptamine inhalation: effects on salivary IL-6, cortisol levels, affect, and non-judgment. *Psychopharmacology (Berl)*. 2019;237:773–85. doi: 10.1007/s00213-019-05414-w 31822925 PMC7036074

[R27] Yeshurun Y, Nguyen M, Hasson U. The default mode network: where the idiosyncratic self meets the shared social world. *Nat Rev Neurosci* 2021;22:181–92. doi: 10.1038/s41583-020-00420-w 33483717 PMC7959111

