# Supplementary material for: Exploring 5-MeO-DMT as a pharmacological model for deconstructed consciousness
Source: Neurosci Conscious. 2025 Apr 21;2025(1):niaf007. doi: 10.1093/nc/niaf007 (PMC12010161; doi:10.1093/nc/niaf007)
Supplement: niaf007_Supp [file niaf007_supp.zip › suppl_data/Deconstruct_5_Sl_revised_clean.docx.docx]

**Supplementary Information for:**

**Exploring 5-MeO-DMT as a pharmacological model of deconstructed consciousness**

Christopher Timmermann*, James W. Sanders, David Reydellet^,^ Tommaso Barba, Lisa X. Luan, Óscar Soto Angona, Genís Ona, Giancarlo Allocca, Carl H. Smith, Zachary G. Daily, Natasha L. Mason, Lilian Kloft-Heller, Martin Kuchar, Lucie Janeckova^,^ Tomas Palenicek, David Erritzoe, Johannes G Ramaekers, Robin L. Carhart-Harris, Malin Vedøy Uthaug

*Correspondence: Christopher Timmermann ([c.timmermann-slater15@imperial.ac.uk](mailto:c.timmermann-slater15@imperial.ac.uk))

**This file contains:** - Supplementary Methods

- Supplementary Results

- Supplementary Figure 1

- Supplementary Tables 1-3

**Supplementary Methods:**

Analysis of toad secretion

Standards of analytes were obtained from commercial suppliers as follows: 5-Methoxytryptophol (5-MeO-tryptophol), 5-Hydroxy-Nω-methyltryptamine (N-Methylserotonin, NMe-5HT) and bufogenin were supplied from Sigma-Aldrich, CZ. 5-Hydroxytryptophol (5-OH-tryptophol) was purchased from Fluorochem Ltd., GB. N,N-Dimethyltryptamine (DMT), 5-methoxy-N,N-dimethyltryptamine (5-MeO-DMT), 5-hydroxy-dimethyltryptamine (bufotenin, 5-OH-DMT) were synthesized and characterized at UCT Prague.

Stock solutions of all analytes were prepared by dissolving exact amount in MeOH/water, 50/50 (v/v) to a final concentration of 1 mg/mL and stored at -20 °C. Working solutions were made by further dilution with MeOH/water, 10/90 (v/v). Calibration ranges were 10 ng/mL – 1000 ng/mL for all analytes. The sample was weighted to a 5mL volumetric flask and dissolved in MeOH/water, 50/50 (v/v) using an ultrasonic bath. Further dilutions of this solution with MeOH/water, 10/90 (v/v) were made by pipetting.

For UHPLC-MS analysis, an UltiMate 3000 LC system (Thermo, USA) consisting of a HPG-3000RS pump, a WPS-3000RS autosampler, and a TCC-3200RS column compartment was used. MS detection of the eluates from UHPLC system was carried out with a QTrap 6500 mass spectrometer (AB Sciex, Canada). The final optimized LC–MS method utilized a ZORBAX Eclipse Plus 95Å C18, 2.1 x 150 mm, 1.8 µm (Agilent, USA) with ZORBAX SB-C18, 2.1 mm, 1.8 µm, UHPLC guard column (Agilent, USA). Mobile-phase A consisted of water with 0.1% formic acid and 5mM ammonium formate, mobile-phase B was pure methanol. The gradient program was as follows: 0–0.5 min, 10% B; 0.5–6 min from 10% B to 100% B; 6–9 min, 100% B; 9–9.5 min from 100% B to 10% B; and equilibration of the column to 12 min. The autosampler temperature was kept at 10°C. The flow rate of the mobile phase was 200 μL/min, and the column temperature was adjusted to 30°C. The injection volume was 2 μL. For data acquisition and evaluation, Analyst software version 1.63 and Sciex OS 2.0.1 were utilized (AB Sciex). A multiple reaction monitoring (MRM) MS method was used for all studied analytes. Final MS conditions were: Turbo-V ion source equipped with ESI probe in a positive mode (ESI+), ion spray voltage was 5350 V, curtain gas was 20 psig, temperature was 450°C, ion source gas 1 was 30 psig, ion source gas 2 was 30 psig.

**Supplementary Results**

Interview Excerpts organised by category

Onset

- “I guess [the first thing that happens is] this rising up feeling… it's located just everywhere… A sense of very much like go... Not going, like changing in position, but moving into some sort of alternative space. Not a... Not like a physical space. Like, I guess it's a mental space.” Participant 12
- “Well, there were some visuals, actually, in the very beginning…. Like, like, as if you're shooting through the universe, interspace, kind of, you know, like patterns and stars or stripes kind of moving very quickly, acceleration, for sure, there was definitely an acceleration feeling.” Participant 7

Immersion & merging

- “And I guess there's, like, head feel as well that's undeniable. Like, this sort of... It's a bit like your mind is being 'zhoomed'. 'Zhoom!'. Like that… And also, like, whitish colours and off-white coloured colours, like white and, you know, there's a sort of colour scheme of like, white and then like, close to white purples close to white blues, close to white. those kinds of colours. Yeah. And I didn't experience those colours. But it was like I was entering into a space that was sort of felt like those colours… I completely forgot about my body… Not that I didn't feel my body, I just- my mind wasn't focused on it at all. [Like] When I'm in my work, I don't focus on my body.” Participant 12
- “it wasn't visual for me. I certainly didn't see anything like colours or anything like that… [I heard] just what I can hear now [the sounds of the room], … [and] I'm kind of thinking 'what if I let go?' … And as I allow myself to go there... Yeah, it’ almost like, maybe it's a bit like it's how the fear is manifesting... It's like... some trapped energy here basically, that's all I can say. It's like I'm partly fighting it, partly letting it out.” Participant 9

Abstract

- “I felt home. It's like basically being infinite with everything... it was shocking to see, like 'okay, so that's it?'… I let myself go and this is where I kind of become one with it, and then that was everything. I felt there was so much, it was so intense for me to...- as if there was like a source of knowledge, or a source of- it was 'the' source, it's like a source of everything, and connecting to that was too overwhelming at the beginning… [and] there was, like, the whole, like, thing going on with like, the fractals and the figures” Participant 9
- “It was just, like, being there. Like, not really consciousness about anything else just than my own existence. Not even with mind, I would say. At that point, at least at the beginning, I wasn't really conscious. I wasn’t thinking, I was just like there, I just sort of stay in the void... I don't know if I would describe it as like navigating through space, but it was, I would say something similar, like wavy patterns… maybe linear patterns. It did have some colours. I wouldn't define what colours, but I could see curvy patterns, and maybe linear at some point. Yeah, but not very well-defined. With perceptions and sensations and emotions that could not be, like, physically localised but they felt like they were happening.” Participant 3

Everything / Nothing

- “All ability to determine any qualities or characteristics just evaporates. So there's only - … I hesitate to use the word awareness because I can't say ‘I’ was aware. But any experience or perception just kind of dissolves, and then nothing takes its place.” Participant 4
- “There's just a sense of being all things. And singular… There's no location to it. It's all, it encompasses everything, [with a] sense of ‘all is right’ Participant 6
- “Then when I was sucked into it, and this is- this is the part where I can't really put into words because.. it felt as like a piece of, like, a puzzle that just, like, was perfectly fitted. Then it just like- it was 'being' at that point, just like, doing whatever the hell that thing is doing. Which was doing nothing actually, and doing everything at the same time… When, I [was sucked in] I don't know what the hell happened anymore. It's just like, you become one with this thing” Participant 9

Reconstitution

- “First thing I felt was this [pressure], because the [EEG] machine was attached to my head… And I'm just like, 'Okay, we're back in meat land, if that's possible again’…. it felt very odd to be back in the body to be back in that room... it felt really, like the energy in the room basically just went [makes rushing noise] and then you're sort of 'okay, we're back here again'.” Participant 8
- “I feel like my mind is taking over again little by little because then I start to have thoughts. So I think during this period before that I didn't have thoughts because then I start to have like thoughts that pop out. Yeah. Like your inner voice, right? Like you are [Person 1] or you are here or this is [Person 2]. This is, you know, like your inner dialogue, right? But at the beginning I think they are not very well connected. I feel quite lost, quite disoriented. And then I start to feel more my body. Like I start to receive more information from my body… I had sensory input coming, you know, like I knew I had a body and I was hearing the music and I was with people and [name] was telling me to breathe. So that's how I knew that I came back.” Participant 10

Afterglow

- “The last bit of my lying down was. Just, like, moving into, kind of just, this, meditation, just 'feel' and just 'be'… A bit like a nice crystal bell has been chiming... Yeah, I can sense my being has like a kind of vibration or what people call it or whatever… The quality of my presence feels different. And [it] does feel like yeah, I've been stretched out.” Participant 13
- “I felt this immense feeling of sadness, and, at the same time, I was so happy to be back. And it was just too much to process. Like, how can I just leave that space and just be here, you know, and like, experience things in a very limited sense? That for me, was very difficult to wrap my head around” Participant 9

**
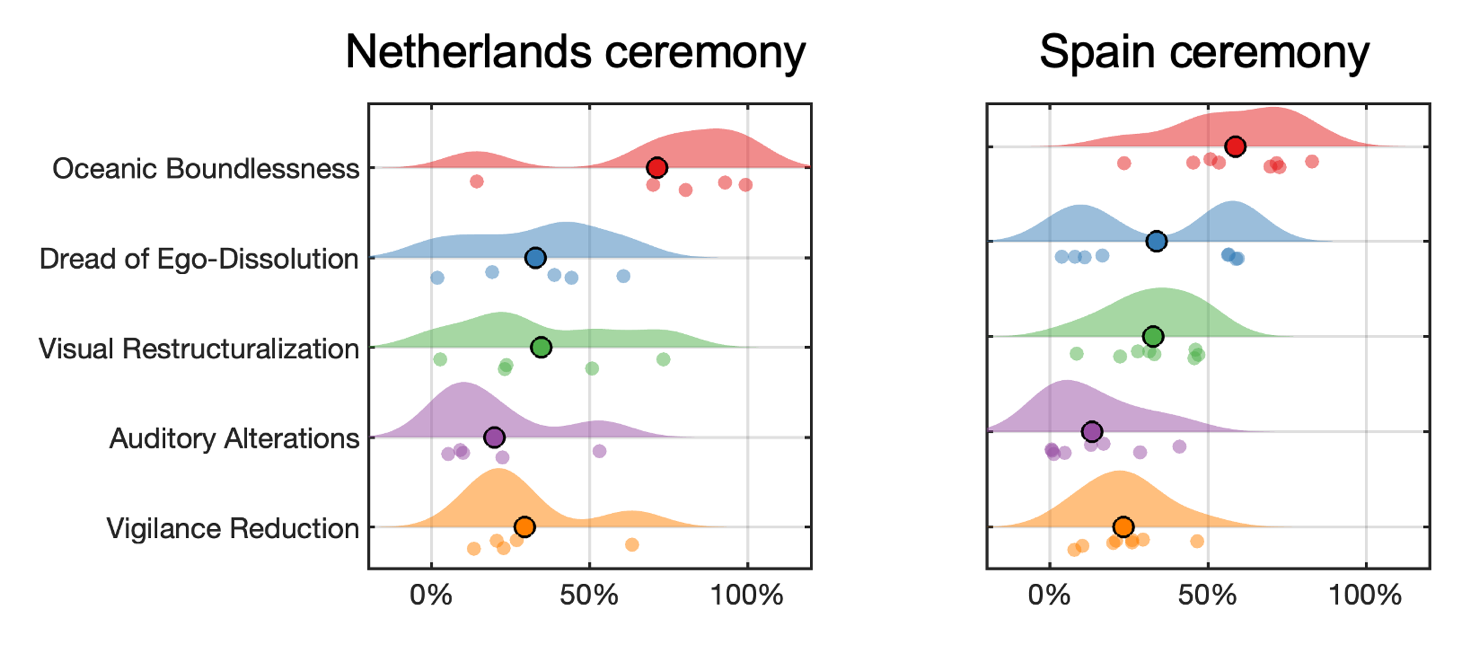
**

**Supplementary Figure 1:** Average scores in the 5-dimension ASC questionnaire for each ceremony separately reveal broadly similar findings despite both ceremonies employing different doses and different formulations of 5-MeO-DMT.

**Supplementary Table 1:** Participant characteristics per ceremony

| **Ceremony** | **Females / Males (%)** | **Age (years)** |
| --- | --- | --- |
| Netherlands | 33.3 / 67.3 | 39 ± 22.6 |
| Spain | 50 / 50 | 34.4 + 5.5 |

**Supplementary Table 2:** Results of analysis of toad secretion (2023). BAFA code 2023-103-VV

| **analyte** | **concentration** | **units** |
| --- | --- | --- |
| 5-MeO-DMT | 262,8 | mg/g |
| Bufotenin | 3,462 | mg/g |
| DMT | 0,031 | mg/g |
| NMe-Serotonin | 0,023 | mg/g |
| Bufogenin | 0,012 | mg/g |
| 5-MeO-Tryptophol | 0,698 | µg/g |
| 5-OH-Tryptophol | ND | - |

*ND:* not detected

**Supplementary Table 3:** The percentage of experiences to which each micro-phenomenologically identified category applied, separated by ceremony group.

| **Category Name** | **Netherlands** | **Spain** | **Total** |
| --- | --- | --- | --- |
| Onset | 83% | 88% | 86% |
| Immersion & merging | 67% | 25% | 43% |
| Abstract | 33% | 50% | 43% |
| Everything/nothing | 17% | 38% | 29% |
| Reconstitution | 33% | 75% | 57% |
| After(glow) | 83% | 13% | 43% |
