# Supplementary figures and images for: Exploring 5-MeO-DMT as a pharmacological model for deconstructed consciousness
Source: Neurosci Conscious. 2025 Apr 21;2025(1):niaf007. doi: 10.1093/nc/niaf007 (PMC12010161; doi:10.1093/nc/niaf007)

Netherlands ceremony

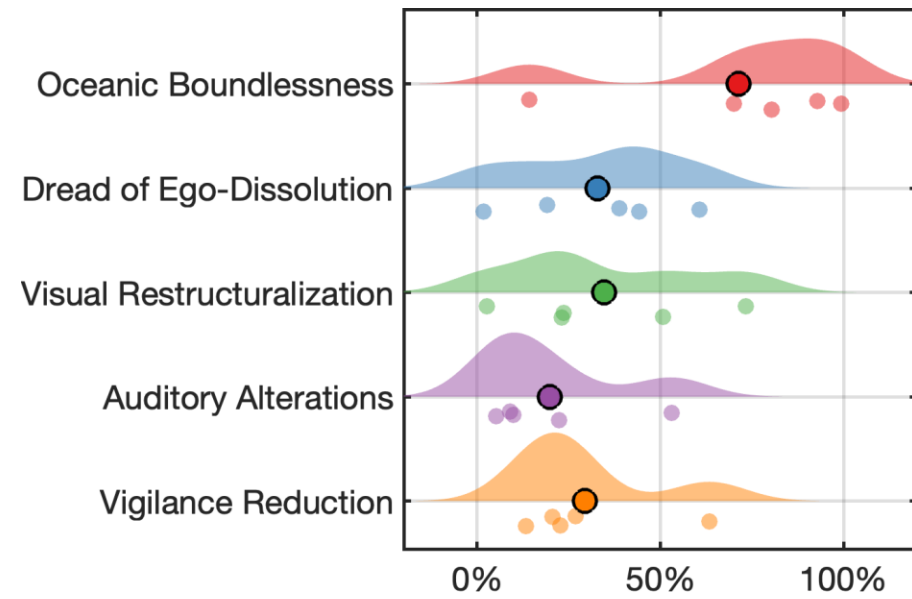

Spain ceremony

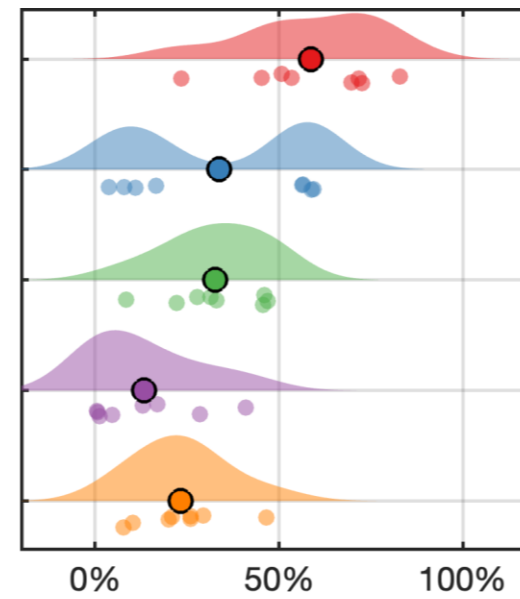

Supplement: niaf007_Supp [file niaf007_supp.zip › suppl_data/FigureS1.pdf]
